# Exploring Physician-Nurse Relations Through the Lens of Intersectionality: A Scoping Review

**DOI:** 10.7759/cureus.83685

**Published:** 2025-05-07

**Authors:** Bianca Nguyen, Dylan Mai, Manuel G Venegas, Pearl Omo-Sowho, Lauren Han, Aishwarya Natarajan, Chidinma Chima-Melton, Anna Dermenchyan

**Affiliations:** 1 Department of Medicine THINQ Collaborative, University of California Los Angeles David Geffen School of Medicine, Los Angeles, USA; 2 Medicine, Burrell College of Osteopathic Medicine, Las Cruces, USA; 3 Medicine, University of California Irvine School of Medicine, Irvine, USA; 4 Medicine, University of Southern California Keck School of Medicine, Los Angeles, USA; 5 Medicine, University of California San Francisco School of Medicine, San Francisco, USA; 6 Medicine, Loma Linda University School of Medicine, Loma Linda, USA; 7 Department of Medicine Quality, University of California Los Angeles David Geffen School of Medicine, Los Angeles, USA

**Keywords:** interdisciplinary communication, interprofessional relations, intersectional framework, patient care team, physician-nurse relations

## Abstract

This scoping review examines how intersectional variables shape physician-nurse relations, addressing a gap in research on how social identities influence collaboration and communication. Using the PROGRESS-Plus framework, we analyzed 21 US-based inpatient studies published between 2000 and 2020, focusing on factors such as age, sex/gender, race/ethnicity, and specialty. Findings revealed that sex/gender and specialty were the most frequently examined variables, while race/ethnicity was notably underrepresented. Although some studies considered multiple variables, most did not intentionally apply an intersectional framework, limiting a comprehensive understanding of how these factors interact. Additional variables, including years of experience, spoken language, and workplace culture, also shaped physician-nurse dynamics. To improve interdisciplinary collaboration, future research should intentionally integrate intersectionality into study designs, medical education, and clinical practice. Addressing these complexities through targeted interventions can foster equitable teamwork and enhance patient outcomes.

## Introduction and background

Effective collaboration and skilled communication between physicians and nurses are essential to hospital operations and patient care. While substantial academic attention has focused on organizational strategies to foster interprofessional collaboration [1], limited research explores the direct role that physicians and nurses themselves play in team dynamics. Previous studies have identified barriers to physician-nurse communication [2], such as structural limitations and contrasting perceptions of teamwork, and proposed strategies, such as redesigning interdisciplinary rounding to improve collaboration [3,4]. However, the influence of social identities, including age, gender, and race/ethnicity, on physician-nurse relations remains underexplored.

Intersectionality, a concept coined by Kimberlé Crenshaw in 1989, describes how overlapping social identities create interdependent systems of discrimination and privilege [5]. Initially used to examine the compounded discrimination faced by Black women [6], intersectionality has since expanded into mainstream discourse and academic inquiry, particularly in studies of health disparities. Despite this growing relevance, its application in healthcare team dynamics is relatively recent. Medical Subject Headings (MeSH) terms are standardized vocabularies used to index articles, ensuring that user searches are comprehensive for a given topic or concept. The National Library of Medicine added "intersectional framework" as a Medical Subject Heading (MeSH) term only in 2022 [7]. While intersectionality has been used to study provider-patient relationships, its role in physician-nurse collaboration remains largely unexplored [8-11].

Understanding how intersectional identities, such as race, class, and gender, shape physician-nurse interactions is critical. These identities influence experiences of power dynamics, marginalization, and conflict within clinical teams [12-14]. Although variables such as age, gender, and race/ethnicity have been identified as predictors of incivility in healthcare teams, results are often inconsistent [12]. Moreover, most studies fail to address the complex interplay of these identities, limiting the depth of insights they can provide.

By applying an intersectional lens to the study of physician-nurse relations, we aim to illuminate barriers to collaboration and identify opportunities for improvement. This scoping review examines existing research on how social identities influence these interactions and highlights the need for more intentional and inclusive medical research. We contextualize findings within the rapidly diversifying field of medicine and propose directions for future exploration to advance equity and teamwork in healthcare.

Conceptual framework 

This study utilizes an adapted version of the PROGRESS-Plus framework, which outlines 16 individual-level variables, including age, sex/gender, race/ethnicity, income, education, employment status, professional status, socioeconomic status/class, marital/partnership status, parental status, single-parent household, immigration status, religion, disability, sexual orientation, region of residence, and urbanity/rurality [15]. Originally developed to assess the impact of social determinants on health inequities, the PROGRESS-Plus framework is also applicable for applying an equity lens to literature reviews.

For this review, we selected age, sex/gender, race/ethnicity, and specialty as focal variables. Our decision was guided by two factors: (1) these variables yielded a higher number of potentially relevant articles (>10) in our searches on physician-nurse relations, and (2) they are the most frequently collected demographic variables in studies, even when not explicitly the study focus. Specialty was included based on evidence demonstrating its influence on physician-nurse relationships and the impact of gender roles on communication within and across medical specialties [16,17].

**Figure 1:**
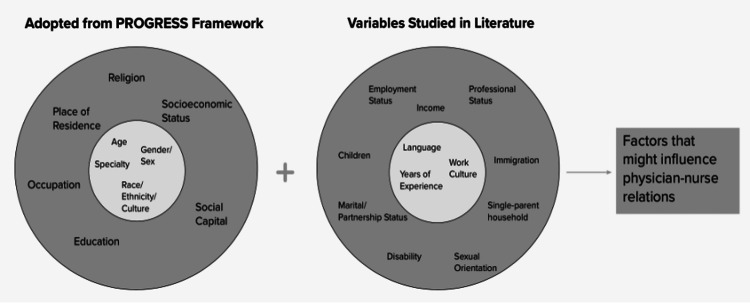
Conceptual model of intersectionality variables that may influence physician-nurse relations The “PROGRESS” framework has been adapted to include socially stratifying variables of interest included and excluded in the scoping review. Within the two darker outer circles is a combination of variables accounted for in the PROGRESS framework and those discovered during the initial literature search. The leftmost inner circle includes the four primary variables based on the PROGRESS framework used in this review. The rightmost inner circle contains three variables later included due to them being frequently cited in the literature. The combination of effects due to individual variables interacting with one another contributes to the complex nature of the physician-nurse relationship. Image credits: Bianca Nguyen, Dylan Mai, Manuel G. Venegas III, Pearl Omo-Sowho, Lauren Han, Aishwarya Natarajan, Chidinma Chima-Melton, and Anna Dermenchyan.

The adapted framework and relationships between these variables are shown in Figure 1. The primary aim of this study was to examine how these individual-level markers, which shape personal experiences, have been addressed in existing research on physician-nurse communication. A secondary aim was to incorporate an intersectional lens to summarize findings on both the individual impact of these variables and their interactions. By doing so, this study identified existing gaps in research methodologies and barriers to effective physician-nurse collaboration. These findings provide a foundation for future studies to develop more inclusive frameworks that enhance interdisciplinary collaboration and promote equity in healthcare settings.

## Review

Methods

Study Design

This scoping review was conducted to explore how intersectionality has been studied within physician-nurse relations and to identify variables that facilitate or hinder these interactions in clinical practice. Rather than evaluating study quality or conducting a meta-analysis, this review aimed to map existing evidence, highlighting key concepts, trends, and gaps in the literature. The review followed the PRISMA Extension for Scoping Reviews (PRISMA-ScR) Checklist, developed in 2018.

Eligibility Criteria

Articles were included if they met the following criteria: they focused on physician-nurse relations, examined at least one of the intersectionality variables (age, sex/gender, race/ethnicity, or specialty), were conducted in inpatient settings, were based in the US, and were published between 2000 and prior to the COVID-19 pandemic. Studies that did not meet these criteria were excluded during database searches, assessments using the inclusion tool, or presentations during the group review process.

Search Strategy

**Figure 2:**
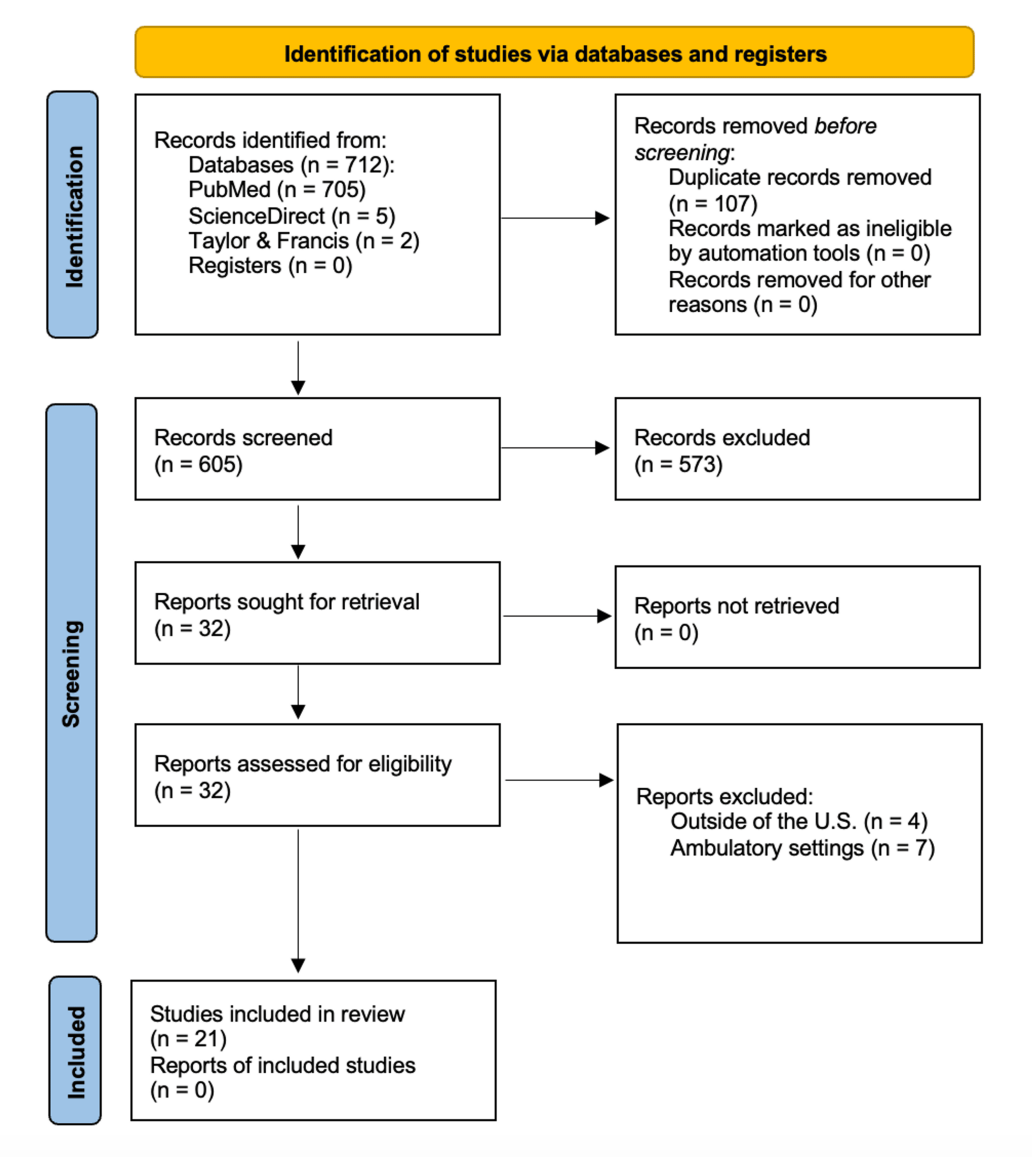
PRISMA flow diagram of study screening and selection. PRISMA: Preferred Reporting Items for Systematic Reviews and Meta-Analyses

The literature search was conducted using PubMed, with “physician-nurse relations” selected as the primary MeSH term to describe the interaction between physicians and nurses. Additional searches were performed in ScienceDirect and Taylor & Francis databases. To ensure comprehensive coverage, intersectionality variables were assigned to groups of two to three researchers for focused investigation. Searches were conducted using various combinations of terms, with the number of articles retrieved recorded for each search. For example, the search strategy for the age variable included the following terms: "Physician-Nurse Relations AND age" OR "Physician-Nurse Relations AND older" OR "Physician-Nurse Relations AND middle-aged". For race/ethnicity, the search strategy was as follows: “Physician-Nurse Relations AND race” OR “Physician-Nurse Relations AND culture” OR “Physician-Nurse Relations AND prejudice” OR “Physician-Nurse Relations AND ethnicity”. This process was repeated for each variable until a sufficient number of relevant articles (>20) was identified (FIgure 2).

Study Selection

The study selection process followed a two-phase screening approach. In the first phase, titles and abstracts were screened for relevance based on predefined inclusion criteria. In the second phase, full-text articles that met the eligibility requirements were reviewed in detail using an adapted assessment tool based on a guide by Souza et al. [18]. Each article was categorized according to its primary intersectionality variable. If a study addressed multiple variables (e.g., age and gender), it was assigned to the most relevant variable group for further analysis. The most relevant variable group was determined by degree of inclusion in the study’s research objectives, analysis, and/or findings.

Data Extraction and Analysis

Data extraction was conducted using a standardized extraction form to ensure consistency across studies. Articles were evaluated based on how intersectionality variables were incorporated, whether interactions between variables were noted, and whether these interactions were intentionally or incidentally studied. Following the initial analysis, the research team convened to discuss article relevance, resolve discrepancies, and reach consensus on final study inclusion. Risk of bias assessment was not included in this review as the purpose was to examine all available evidence existing on this topic rather than assess for study quality or make specific clinical recommendations.

Results

A total of 21 articles met the inclusion criteria for this scoping review. These studies examined physician-nurse relations and assessed at least one intersectionality variable (age, sex/gender, race/ethnicity, or specialty) in the context of physician-nurse communication. All included articles focused on inpatient settings in the US. Articles that did not meet these criteria were excluded during database searches, screening using the inclusion tool, or the group review process (Figure 2). The final set of articles was published between 2001 and 2020 (Table 1).

**Table 1:** Summary of the literature included in the scoping review of physician-nurse relations through the lens of intersectionality Twenty-one articles were analyzed for “Exploring Physician-Nurse Relations Through the Lens of Intersectionality: A Scoping Review." The table highlights the citation, design/sample, purpose/results, and intersectionality findings. Column A shows the Unintentional inclusion of two or more intersectionality categories. For this, the researchers counted the number of times the article had analyzed and discussed at least two intersectional categories, even if not initially part of the aim of the study. Column B shows the Intentional inclusion of two or more intersectionality categories. For this, the researchers counted the number of times the article had purposefully explored and analyzed at least two intersectional categories in relation to the aim of the study beyond the demographics.

Article information			Intersectionality findings		
Citation	Design/sample	Purpose/results	Summary	A	B
Hoonpongsimanont et al., 2018 [19]	Survey (n = 288 participants)	Assess among four generations if there is consensus on the definition of emergency medicine physician professionalism. Participants included ED nurses, physicians, residents, and medical students. The majority (70.49%) were millennials and predominantly female (>50%). Millennials and Generation X both ranked respect for patients as the most important quality in emergency physician professionalism (EPP). In the baby boomers and the silent generation, no consensus on professionalism was significant.	Age and gender affected the consensus of attitudes on emergency department professionalism.	0	0
Nath et al., 2006 [20]	Survey (n = 596)	Evaluate if the perceptions of professionalism vary with age, discipline, gender, or educational level. Younger individuals (age <26) were significantly more likely to rank behaviors such as "whispering in class" as unprofessional than other age groups. Those in the oldest group (>51) were more likely to rank statements that reflected honesty and responsibility as unprofessional. The statement "physician calling in sick" had the biggest contrast between the <26 and >51 age groups in its relation to professionalism. Nurses and doctors with larger age gaps may hold stereotypes against each other based on their professional views. Females were more likely than males to rank a behavior as unprofessional when gender differences were found.	Age and gender, to a lesser degree, may have affected how people in healthcare view the professionalism of different tasks. Similarly, educational level may have impacted the perception of professionalism, while the healthcare discipline may have played a minor role.	1	1
Siedlecki et al., 2015 [21]	Survey (n = 1364)	Study nurse and physician perceptions of their relationships within the professional environment regarding respect and its effects on nursing practice decisions. Most nurses were female (n = 732; 95%), and most physicians were male (n = 372; 72%). Physicians rated the overall environment, physician characteristics, and patient care decision-making more highly than nurses. Nurses were more likely than physicians to value the impact of respect, communication, and collaboration on patient outcomes. A significant difference in the mean levels of years of professional experience. Younger (age < 45) and less experienced (< 20 years) nurses were more likely to be affected by negative physician behaviors.	Age, gender, and years of experience may have affected perceptions of the physician-nurse relationship.	1	1
Brewer et al., 2013 [22]	Survey (n = 1328)	The study examined the relationship between physician verbal abuse of new registered nurses in relation to demographics, work attributes, and perceived professional environment. Positive correlations were found with hospital setting (p < 0.001), direct care position (p = 0.002), 12-hour shift (p < 0.001), daytime schedule (p < 0.001), voluntary overtime (p = 0.002), and mandatory overtime (p = 0.001). English as a first language (p = 0.003) and White Hispanic/non-Hispanic ethnicity (p = 0.004) were generally negatively associated with verbal abuse. RNs experiencing moderate abuse were significantly younger than those with no abuse (p < 0.001). No association was found between verbal abuse and gender.	Ethnicity and age were factors associated with physician verbal abuse, while gender was not. Significant associations existed between physician verbal abuse and the variables of language, work culture, employment, and education. No association existed between children and marital status.	1	1
Espeut et al., 2020 [23]	Questionnaire (n = 312)	Analyze the perception of nurses of all ages and years of experience regarding women surgeons. 54% of nurses agreed with the statement, "Female surgeons interact differently with nurses when compared to male surgeons," with those with more experience in nursing being more likely to agree. Nurses with more experience were less likely than their counterparts to agree that women made good surgeons (p = 0.01) and were as capable as male surgeons (p = 0.02). As age increased, 44% of nurses believed women surgeons had the same advancement opportunities as men (p = 0.01).	Gender, age, and experience were associated with nurses' perception of women surgeons.	1	1
Hojat et al., 2001 [24]	Survey (n = 639)	Study the attitudes of U.S. and Mexican nurses and physicians on team collaboration. The gender of physicians was not found to be a significant variable affecting attitude. U.S. samples expressed more positive attitudes toward collaboration than Mexican samples. Nurses expressed more positive attitudes toward collaboration than physicians. There was a link between age and attitudes only for U.S. physicians, but not for the other groups.	Gender was not associated with physician and nurse attitudes on team collaboration. Age, region of residence, professional status, and organizational culture were associated with attitudes.	1	1
Lyndon et al., 2011 [25]	Survey (n = 125)	Positive relationships existed between obstetric clinicians' assessment of role-specific communication quality and reported likelihood of speaking up about potential patient harm. A negative relationship existed between self-reported exposure to disruptive behaviors and the likelihood of speaking up. Nurses attributed higher ratings for potential harm in scenarios (p < 0.001). They reported higher exposure to disruptive behaviors (p = 0.002) and lower teamwork climate (p = 0.046) than physicians. Physicians reported higher work stress scores (p < 0.001). Likelihood of Speaking Up score positively correlated with scores of bravery and assertiveness (p < 0.05). Bravery and assertiveness scores were positively associated with age (p < 0.05) and years of experience (p < 0.001). Harm Index score associated with Likelihood of Speaking Up Index score (p = 0.002).	Age and years of experience affected the likelihood of speaking up among nurses and obstetricians.	1	1
Rothstein et al., 2007 [26]	Questionnaire (n = 125)	Analyze nurses' perceptions of male and female physicians based on profession and gender. Nurses rated female physicians similarly to male physicians, but still more favorably on all questions in the questionnaire. Both male and female physicians were rated favorably on topics related to patient care and less favorably on those about physicians' sensitivity to the nurses' administrative responsibilities. Nurses in the study were least satisfied with physicians' recognition of their other responsibilities besides caring for their patients. Nurses rated younger female physicians (< 50 years old) more favorably than older female physicians on all questions, but this data could not be tested.	Physician-nurse relationships were influenced more by profession than gender, with age a potential confounder. These findings considered the higher education level and experience of advanced practice nurses.	1	1
Brucker et al., 2019 [27]	Evaluation (n = 1112)	Assess nursing evaluations of female emergency medicine physicians to evaluate gender bias. Female residents reported being less professional in interactions with nurses (p=0.041) and having less effective team leadership skills (p = 0.019) than their male counterparts. Nurses are less likely to report being comfortable with female residents caring for their family members (p=0.013). 51% of female residents had negative ability (e.g., technical skill, knowledge, competence) comments, and 57% had negative grindstone (e.g., work ethic, effort, efficiency) comments. 30% of ability comments about female residents appeared tentative, while only 14% of ability comments about male residents were tentative (p<0.01).	Gender-affected nursing evaluations of emergency medicine residents are inconsistent with examination scores and milestone evaluations.	1	1
DeKeyser et al., 2016 [28]	Ethnography (n = 42 interviews; n = 81 survey)	A model to describe ICU interprofessional shared clinical decision making and the factors associated with its implementation. Shared clinical decision making occurs on four stepped levels. Nurses in the U.S. had active and defined roles during morning rounds. Physicians conduct one-way communications rather than engaging in cooperative interactions. Nurses and physicians with more experience were involved more than their new counterparts in shared decision making. Gender was reported as a barrier to shared decision making.	Gender may have influenced the level of shared decision-making between ICU nurses and physicians. Professional status, region of residence, work culture, and experience may have also impacted this relationship.	1	1
Galvin et al., 2015 [29]	Questionnaire (n = 2202)	Study the presence of gender bias in nurse evaluations of obstetrics and gynecology residents. Nurses use harsher language to describe women compared to men. When controlling for confounders, only PGY-2 evaluations contained lower mean ratings for women (p=.001). PGY-1 women received significantly fewer positive and more negative agentic comments than PGY-1 men (p=0.041). Residents who regularly receive destructive or no feedback have poorer learning outcomes, lower examination scores, less confidence in skills upon graduation, and lower competence levels than residents who receive regular, constructive feedback.	Gender affected nursing evaluations of physicians in obstetrics and gynecology. These findings considered the residency education of the physicians evaluated in the study.	1	1
Taylor, 2009 [30]	Survey (n = 304)	Compare attitudes toward collaboration of anesthesiologists and nurse anesthetists. The study failed to show an influence of gender (p=0.449) or the interaction of gender and discipline (p=0.553) on collaboration scores between anesthesiologists and nurse anesthetists. A significant effect of provider discipline on attitude toward collaboration was shown (p=0.000). A positive correlation between attitude toward collaboration and years of anesthesia experience was found in anesthesiologists (p=0.031), and a negative correlation was found in nurse anesthetists (p=0.043)	The effects of gender and the interaction of gender and discipline were not significant. Years of anesthesia experience were associated with attitudes toward collaboration among anesthesiologists and nurse anesthetists.	0	1
Wear et al., 2004 [31]	Interview (n = 51)	Examine attitudes of female physicians and female nurses in surgery, internal medicine, obstetrics-gynecology, and emergency medicine. Gender was considered more important than occupation in female nurses' perceptions of the relationship, while occupation was considered more important than gender in female physicians' perceptions of the relationship. Nurses generally expressed more satisfaction in their relationships with female physicians. Female residents' most common problems with female nurses were related to receiving less assistance and challenges in being perceived as more demanding. While positive sentiment was generally expressed in all departments, more amicable relationships between female nurses and female residents were emerging in the surgery and emergency medicine departments of obstetrics-gynecology.	Gender and specialty may have influenced how residents and nurses perceive each other. Professional status interacted with gender to inform attitudes.	1	1
Robinson et al., 2019 [32]	Focus group (n = 18)	Identification of themes for effective and ineffective communication. Effective communication themes include clarity and precision of message that relies on verification, collaborative problem solving, calm and supportive demeanor under stress, maintenance under stress, and authentic understanding of the unique professional role. Ineffective communication themes include making someone less than (derision), dependence on electronic systems, and linguistic and cultural barriers.	Differences in culture potentially hindered good communication. Poor communication was associated with those whose first language was not English. Salient factors such as tone and preconceived assumptions about cultures may impact communication.	1	0
Brinkman et al., 2006 [33]	Evaluation (n = 332)	Evaluate if parent and nurse ratings significantly differ from attending physician ratings of pediatric residents. Nurses rated residents lower than did attending physicians on categories of respecting staff, accepting suggestions, teamwork, being sensitive and empathetic, respecting confidentiality, demonstrating integrity (all p < 0.01), and demonstrating accountability (p < 0.02). Nurses rated residents higher than attending physicians on anticipating post-discharge needs (p < 0.01) and effectively planning care (p < 0.03).	Nurses rated residents lower on multiple teamwork factors than attending physicians in the pediatric setting.	0	0
Cook et al., 2001 [34]	Questionnaire (n = 78)	Negative relationship with the physician was the most severe long-term effect of verbal abuse identified. Abusive anger is determined as the most frequent and stressful type of abuse. Frustration and anger are the strongest emotional feelings identified by nurses in reaction to verbal abuse. 91% of nurses reported experiencing at least one episode of physician verbal abuse in the past. The most frequent long-term adverse effects are in relationships with physicians, job satisfaction, sense of relaxation/being the main in the workplace, trust and support in the workplace, and self-esteem.	Perioperative nurses experienced frequent verbal abuse behaviors from physicians.	1	0
Foronda et al., 2019 [35]	Simulation (n = 8)	Assess the reliability of the Identification, Situation, Background, Assessment, and Recommendation (ISBAR) Interprofessional Communication Rubric (IICR) among graduate pediatric nurses and inspect their communication performance scores. The ISBAR IICR may be a reliable tool to help train and evaluate communication patterns and measure performance. Median communication score was 7.3 out of 15 points, suggesting a need for remediation as the average score was less than a passing score.	New and less experienced pediatric nurses identified communication and interprofessional development areas for improvement in collaboration with other team members.	1	0
Makary et al., 2006 [36]	Questionnaire (n = 2135)	Compare teamwork ratings within and among operating room caregivers. Implementing OR briefings and debriefings to improve teamwork and communication in the OR can lessen differences in teamwork perceptions, with the goal of improved performance and safety. OR Nurses received the highest rating in teamwork ratings (p < 0.001), followed by surgical technicians (p < 0.001), CRNAs (p < 0.001), anesthesiologists (p < 0.001), and surgeons (p < 0.001). Surgeons and anesthesiologists rated teamwork within their discipline the highest and were more satisfied with physician-nurse collaboration.	In the OR, discrepancies existed between physician and nurse perceptions of teamwork.	0	0
Reeves et al., 2014 [37]	Ethnography (n = 56)	Analyze variables that impact interprofessional collaboration and family involvement in care delivery processes. In the ICU, interprofessional interactions between nurses and physicians were brief. Information technology encouraged parallel work practices and separateness observed between medical and nursing activities. Relationships between staff transition to being highly collaborative during periods of urgency or crisis.	ICU staff tended to view teamwork as a profession-specific rather than an interprofessional activity. Work culture had an impact on these views.	0	0
Simpson et al., 2006 [38]	Focus group (n = 92)	Describe communication between labor nurses and physicians within the nurse-managed labor model in community hospitals and its relationship to teamwork and patient safety. Physicians and nurses shared the common goal of a healthy mother and baby, although dissent existed regarding how to achieve this. Fetal assessment and oxytocin administration were two cases of strong disagreement between physicians and nurses. Physicians viewed the existing state of teamwork more positively than did nurses. Physicians tended to preserve hierarchy.	Physicians and nurses had positive views of each other and their interactions in obstetrics. Physicians tended to prefer nurses with more experience.	0	0
Thomas et al., 2018 [39]	Survey (n = 1262)	Explore the effects of nurses' educational backgrounds and clinical specializations on nurses' status affirmation by physicians. Highly educated nurses are less likely to receive status affirmation from physicians. Nurses who work in frontline patient care are more likely to receive status affirmation from physicians. Higher education of nurses does not garner more respect or positive interactions from physicians.	Specialty had an impact on nurses' perceived status affirmation. Professional status plays a part in affirmation, while education does not.	1	0

Study Selection and Inclusion by Variable

The search for the age variable yielded 74 articles, of which eight met the inclusion criteria after screening [19-26]. The sex/gender variable produced 504 articles, with 12 included after full-text review [19-24,26-31]. The race/ethnicity variable returned 40 articles, but only two met the inclusion criteria [22,32]. The specialty variable search identified 94 articles, with 14 included in the final review [23,25,27-31,33-39]. Although a total of 21 unique articles met the inclusion criteria, some studies addressed multiple variables, resulting in a total of 36 variable-specific analyses (Table 2). 

Study Characteristics

Most studies utilized quantitative methodologies, with surveys (n = 8, 38%) and questionnaires (n = 5, 24%) being the most common data collection tools. The majority of studies (n = 15, 71%) did not explicitly use a theoretical framework to guide their analysis. While 15 studies (71%) mentioned two or more intersectionality variables in their findings or discussion, only 12 (57%) intentionally examined multiple variables in their research design. The number of intersectionality variables analyzed per article ranged from 1 to 3, with an average of 1.7 variables per study (Table 2).

**Table 2:** Four selected intersectionality variables studied in the literature A bolded X (X) indicates the main variable in which the study/article was categorized. Age is the years between a person’s birth and when the specified study was conducted. Sex/gender: Sex is defined as the biological assignment of male, female, or intersex at birth, determined by the interaction of sex hormones and associated with reproductive functions. Gender, by contrast, is an individual’s self-identification as male, female, or another gender identity. It is a socially and culturally constructed term highlighting differences between male and female characteristics in society that exist on a continuous spectrum. While the authors recognize that sex and gender are not dichotomous, many articles in this study treat these terms as such in their study design. Race/ethnicity: Race is a category of humankind that shares certain distinctive physical traits such as skin color, facial features, and stature. Ethnicity refers to the identification of a group based on a perceived cultural distinctiveness that makes the group a “people.” The authors of these studies use race and ethnicity interchangeably. Specialty refers to a medical specialty, a specific branch or area of medicine with defined patient populations, diseases, and skills. A single hyphen (-) in a cell indicates that no data were reported or the variable was not examined in the respective study.

Citation (n=21)	Age	Sex/ Gender	Race/ Ethnicity	Specialty	Total Variable Count per Article
Hoonpongsimanont et al., 2018 [19]	X	X	-	-	2
Nath et al., 2006 [20]	X	X	-	-	2
Siedlecki et al., 2015 [21]	X	X	-	-	2
Brewer et al., 2013 [22]	X	X	X	-	3
Espeut et al., 2020 [23]	X	X	-	X	3
Hojat et al., 2001 [24]	X	X	-	-	2
Lyndon et al., 2011 [25]	X	-	-	X	2
Rothstein et al., 2007 [26]	X	X	-	-	2
Brucker et al., 2019 [27]	-	X	-	X	2
DeKeyser et al., 2016 [28]	-	X	-	X	2
Galvin et al., 2015 [29]	-	X	-	X	2
Taylor, 2009 [30]	-	X	-	X	2
Wear et al., 2004 [31]	-	X	-	X	2
Robinson et al., 2010 [32]	-	-	X	-	1
Brinkman et al., 2006 [33]	-	-	-	X	1
Cook et al., 2001 [34]	-		-	X	1
Foronda et al., 2019 [35]	-	-	-	X	1
Makary et al., 2006 [36]	-	-	-	X	1
Reeves et al., 2014 [37]	-	-	-	X	1
Simpson et al., 2006 [38]	-	-	-	X	1
Thomas et al., 2018 [39]	-	-	-	X	1
Total Variable Count Per Category	8	12	2	14	Average Count: 1.7

Discussion

In selecting variables from the PROGRESS-Plus framework to study intersectionality in physician-nurse relations, we observed that many of the articles included in our scoping review focused on broad, overarching factors influencing physician-nurse relationships rather than intentionally studying specific identity-related variables. A majority of the studies identified that variables such as age, sex/gender, ethnicity/race, or specialty might influence the physician-nurse relationship, but these conclusions were often drawn only after the study was completed. Although our focus was on these four specific variables, other relevant emergent variables were identified during the review process. These variables, although not the primary focus, appeared frequently in the studies we reviewed, suggesting that they may also impact physician-nurse relations and could provide useful context to individual identities.

*Age* 

An emergent variable often discussed alongside age was years of professional experience. The literature revealed that the effect of age and years of experience on the physician-nurse relationship was not uniform. However, significant differences were more commonly attributed to age than years of experience. Articles that highlighted age-related differences typically focused on issues like discrimination, verbal abuse, and perceptions of professionalism in the workplace [19,20,22]. By contrast, studies linking years of experience to physician-nurse relations often focused on perceptions, attitudes, and levels of assertiveness [21,23,25].

*Sex/Gender* 

 While no specific emergent variables were found in conjunction with sex/gender, the distinction between sex and gender as separate social categories was often unclear in the literature. Many studies did not differentiate between sex and gender or asked participants to indicate only one of these categories. Studies on the relationship between sex/gender and physician-nurse relations were divided on whether gender was a significant factor. Articles that found no association between gender and physician-nurse relations often focused on verbal abuse, collaboration attitudes, and shared decision-making [22,24,26,30]. On the other hand, studies that identified an association with gender measured physician-nurse attitudes, as well as nurse evaluations of physicians [20,23,27-30]. A sub-theme emerged where female physicians were often perceived and evaluated more negatively than their male counterparts in terms of knowledge, competence, and professional ability [23,27,29]. This pattern was particularly evident in evaluations by female nurses, who expressed more negative perceptions of female physicians compared to male physicians.

Race/Ethnicity

There was a notable lack of academic focus on race and ethnicity as variables influencing physician-nurse relations, with only one study directly addressing this issue. Brewer et al. (2020) found that nurses who identified as White Hispanic/non-Hispanic and those who spoke English as their first language were less likely to report experiencing verbal abuse from physicians [22]. Language frequently emerged as a key variable in studies examining race and ethnicity, with cultural and linguistic differences often cited as contributors to miscommunication between physicians and nurses. Although race and ethnicity were infrequently studied directly, these findings highlight the importance of addressing language and cultural barriers to improve collaboration and communication in healthcare teams.

Specialty

Specialty-specific factors emerged as an important theme in the literature, with a wide range of specialties represented, including OB-GYN, surgery, pediatrics, critical care, emergency medicine, and anesthesiology. There was no uniform consensus on how specialty influenced physician-nurse collaboration; rather, the extent of collaboration varied across and within specialties. For example, OB-GYN and emergency medicine specialties typically offered more opportunities for physician-nurse interaction [25,29,31,38]. By contrast, ICU settings, where roles and tasks are more clearly defined, showed that interactions between physicians and nurses were less frequent and often negatively influenced collaboration when they did occur [28,37]. One study found that physicians in roles with more specific task definitions, such as those in surgery and anesthesiology, rated their collaboration with nurses more positively than physicians in less-defined roles [36].

Areas of Alignment and Limitations

This review identified common intersections of identity, particularly between sex/gender and specialty, as well as between sex/gender and age. In OB-GYN, female nurses expressed more negative perceptions and interactions with female physicians compared to male physicians. The intersection of sex/gender and age was primarily due to both being common demographic variables collected in studies, with few studies intentionally analyzing their intersection. In many cases, intersections were discovered after the study was concluded or as a byproduct of the study design. For example, studies that sought to evaluate gender bias often inadvertently focused on a particular department, such as the emergency department or ICU, leading to incidental intersections between gender and specialty.

While common themes regarding barriers to physician-nurse collaboration were identified, generalizing the findings is challenging due to several methodological limitations. Many studies had small sample sizes and were conducted within one hospital or specific departments, limiting their external validity. Qualitative study methods and measures, such as convenience sampling, surveys, interviews, and focus groups, introduced potential biases, particularly social desirability bias. Additionally, the lack of uniformity in defining or measuring physician-nurse collaboration, such as through focus groups or ratings, made it challenging to draw comprehensive conclusions. Some studies solely investigated the perceptions of nurses or physicians rather than both. As a result, the selected articles may not fully represent the complexities of physician-nurse relationships.

Despite these limitations, the concept of intersectionality remains a powerful lens through which to understand the complex nature of physician-nurse relationships. While nine out of 21 studies did not attempt to study the impact of multiple variables, the integration of an intersectional framework is essential for understanding the nuanced interactions between different social categorizations. Future research can benefit from employing an intersectional framework from the outset to study how multiple variables intersect to influence physician-nurse relations, offering clearer insights into the dynamics of these relationships.

*Implications* 

This study highlights a significant gap in the intentional application of intersectionality theory within medical research, particularly concerning physician-nurse relations. Although intersectionality theory is widely accepted in academia, its integration into medical research remains limited. Many studies that examined multiple variables did not do so from an explicitly intersectional perspective, undermining the potential for a deeper understanding of how these variables interact to shape outcomes in healthcare settings.

To promote the use of intersectionality in medical research, it is essential for educational programs to integrate intersectional frameworks into their curricula. For instance, disciplines like epidemiology that already incorporate sociological perspectives can benefit from deeper discussions on intersectionality. Furthermore, increasing awareness of intersectionality in clinical practice - particularly in interprofessional collaboration - can help foster positive, equitable relationships among healthcare providers.

In clinical settings, starting with practices like interdisciplinary rounds can provide a platform for healthcare teams to engage in shared decision-making and discuss how individual identities impact patient care [4,40]. However, to truly benefit from interdisciplinary collaboration, healthcare teams must foster an environment where these conversations are welcomed and diverse perspectives are valued. One way this is being addressed in early professional development is through the utilization of nurses as teachers and preceptors to medical residents. Statistically significant increases in physician perception of communication, collaboration, and understanding of nurses’ roles have been demonstrated, as well as positive attitudes from nurses on teaching residents [41,42]. The incorporation of secure text messaging has also been shown to improve physician-nurse communication effectively, with the use of mobile applications such as Voalte being highly predictive of interprofessional bedside rounding occurrence [43]. Another study demonstrated a 59% reduction in potential communication failures following the introduction of secure messaging using quality improvement methods [44]. Through targeted education, research, and practice, healthcare professionals can begin to understand the complex dynamics at play in physician-nurse relations and improve team-based care.

This review focused on studies published between 2000 and 2020 to provide a comprehensive understanding of physician-nurse relations through an intersectional lens before the transformative impact of the COVID-19 pandemic. The selected timeframe reflects a period in which awareness of intersectionality in healthcare was emerging yet not widespread. However, the pandemic highlighted significant inequities in healthcare systems, particularly concerning race, ethnicity, and other intersecting social identities, bringing these issues to the forefront of medical discourse. Given the heightened attention to social justice and systemic inequities during and after the pandemic, future research should examine studies published post-2020 to explore how these dynamics have influenced physician-nurse relations. Recent literature exploring physician-nurse collaboration after the pandemic has so far shown increased positive attitudes, communication openness, and job satisfaction from both physicians and nurses, despite increased physical and mental burden [45, 46]. These findings differ from previous studies, such as those evaluated in this current study, which depict a more strained relationship between physicians and nurses. One hypothesis is that the nature of the pandemic not only required more consistent communication between team members but also united them in a common sense of purpose. This resulted in increased recognition of roles and engagement of nurses in rounds [45]. While no significant effects by gender and specialty were found in one study, interestingly, the youngest age group (20-29 years old) had a significantly more positive response to the statement that “Doctors should be the dominant authority on all health matters” [46]. Whether this perspective is a continuation of generational differences or a byproduct of the pandemic remains to be explored. Investigating the evolution of intersectionality in interdisciplinary collaboration during this period will offer valuable insights into addressing structural inequities and improving team-based care in increasingly diverse healthcare settings.

## Conclusions

While physician-nurse relations have been widely studied to improve collaboration and patient care, research incorporating an intersectional lens remains limited. This review highlights the lack of intentionality in examining how factors such as age, sex/gender, race/ethnicity, and specialty interact to shape these dynamics. Most studies analyzed these variables in isolation, with sex/gender and specialty receiving the most attention, while race/ethnicity was often overlooked or conflated with language and cultural differences.

To advance understanding, future research should deliberately apply intersectional frameworks to explore how multiple social and structural factors influence physician-nurse relations. Intentional study design can generate insights that inform medical education, clinical practice, and policy, fostering more equitable and collaborative healthcare environments.
